# NEWS

**DOI:** 10.7189/jogh.06.010201

**Published:** 2016-06

## AFRICA

➢ In the wake of Ebola and the resultant 11 300 deaths, world leaders are reflecting on its lessons that could prevent, or at least lessen the impact of, the next epidemic. To date, the focus has been on treatment and vaccines, with an announcement at the World Economic Forum on an experimental Ebola vaccine. However, experts warn that this policy – albeit vital, and in line with trends towards supporting targeted health initiatives – risks undermining more crucial, and less funded, efforts to improve basic health infrastructure. Whilst it is easy to demonstrate the impact of targeted initiatives on specific diseases, it is harder to measure the impact of systematic and more low–key initiatives on basic health care, eg, more clinics, better supplies of basic equipment, improved training and diagnosis – and donors like impact. Moreover, more people died of other causes during the Ebola outbreak, but the frightening nature of Ebola gives it a higher profile than more insidious diseases like malaria and diarrhoea. (*International Business Times*, 23 Jan 2016)

➢ Mr Jakaya Kitwete, Africa United Global Ambassador and former president of Tanzania, has called on all African governments to prioritise access to vaccination across the continent. Africa United is an African–wide innovation led by GAVI, the Confederation of African Football, the African Union, the World Bank and the CDC Foundation, and will use sport as a catalyst on critical health issues facing Africa. Mr Kikwete spoke at his formal acceptance as Champion for Immunisation and Global Ambassador, and ahead of the launch of Africa United’s universal immunisation initiative in Kigali, Rwanda. “In Kigali, my esteemed partner in this fight, CAF, will remind everyone that ‘every shot counts’ as we look to achieve our goal of universal immunisation,” he said. (*allafrica.com*, 2 Feb 2016)

➢ 15% of Zimbabwe’s population is HIV–positive, giving it one of the world’s highest prevalence rates, and more than 1.3 million people are living with the virus. In 2013, the Global Fund gave a US$ 555 million grant to enable antiretroviral treatment for Zimbabweans, and currently 700 000 people access treatment. However, these efforts are being seriously undermined by Zimbabwe’s drought, which has reduced its maize harvest from the required 1.2 million tonnes to 200 000 tonnes, and up to 2.4 million people are suffering from food insecurity. Nutrition and HIV are closely linked, as poor nutrition can damage the immune system and accelerate the development of full–blown AIDS. Antiretroviral drugs should be taken on a full stomach, so if people forego meals they could be forced to stop drug therapy – risking the virus mutating into a drug–resistant version. Poor nutrition also renders people more vulnerable to opportunistic infections like tuberculosis. The UN’s Food and Agricultural Organization calls for “vigorous efforts to achieve and maintain good nutrition among HIV–infected people.” Zimbabwe’s National AIDS Council is not tasked with providing additional nutrition to HIV–positive people; and in an attempt to avert catastrophe, the government has acquired 650 000 tonnes of maize for national consumption. (*IPS*, 1 Mar 2016)

➢ In March 2016, South Africa became the first middle–income country to fund social impact bonds (SIBs) for maternal and early childhood development. SIBs are a funding mechanism that draws on upfront capital from investors for services, and a government agency repays the investors, based on outcome performance. These impact bonds were also a first for Africa. Studies of SIBs in other countries have found that they are suited for interventions that have high potential returns (but learning, adaptability and service combinations are needed to realise them), and for interventions that are not core government–funded. Linking payment to outcomes can encourage more government investment in early childhood development; it supports performance management and adaptability; and helps develop the knowledge base of the most effective early childhood development interventions. More countries will launch SIBs in the coming years, and it is important to share lessons to improve the efficiency and quality of their implementation. (*Brookings*, 6 Apr 2016)

➢ In a blog published in the *Financial Times*, Tom Kariuki of the African Academy of Sciences argues that although most African countries face similar health and developmental challenges, their researchers work separately, thus wasting limited human resources and infrastructure. He condemns this lack of collaboration, which meant that eg, lessons from earlier Ebola outbreaks in Uganda and the DR of the Congo could not be shared with Guinea, Liberia and Sierra Leone, as well as competing for a small pool of funding. He calls for the pooling of human resources and more career opportunities for researchers, to halt Africa’s loss of 20 000 scientists each year to developed countries. Funders should promote pan–African collaboration, such as the Developing Excellence in Leadership, Training and Science Africa Initiative (DELTAS). DELTAS supports large networks and consortiums, which address emerging, infectious and non–communicable diseases. The African Union should lobby for more government funding and improve Africa’s R&D expenditure – currently 1.3% of the global total. This should provide the resources for Africa to deal with its problems before they spiral out of control or spread globally. (*Financial Times*, 9 May 2016)

## ASIA

➢ Michael Gideon Marmot, the President of the World Medical Association, highlighted how Thailand’s universal health programme is a model for other emerging economies in Asia in its provision for low–income workers. The programme was introduced in 2002, and was rapidly extended to 18 million uninsured people, with an additional 29 million people covered by less comprehensive schemes. In Thailand, 11% of health care is met by out–of–pocket payments, compared to 63% in India – depriving Indian people of access to health care. However, there are cost constraints within Thailand’s health system, resulting in the government facing pressure to abolish the US$ 0.80 co–payment programme, although there is a great deal of popular support for Thailand’s health system. (*Voice of America*, 9 Feb 2016)

➢ Increasing rates of HIV infection in the Philippines are going against the global trend of decline, with rates of new infection increasing by more than 25% from 2001 to 2011. In 2015, there were 30 356 recorded cases, with more than 80% occurring since 2010, leading to an estimated number of 133 000 recorded cases by 2022. Higher infections amongst injecting drug users (partially due to banning the provision of clean needles without a prescription), coupled with low condom use and high fertility rates have raised fears of “downstream” HIV infections to groups who are not generally at risk, such as children being infected through mother–to–child transmission. Women are often infected through drug–using partners, and are deterred from seeking follow–up exams or ART – which could prevent mother–to–child transmission – due to the stigma surrounding an HIV diagnosis. To offset this, the Sotto Treatment Hub has a designated nurse who tracks women from when they test positive to when they give birth and their babies are tested for HIV. (*Thomson Reuters Foundation*, 15 Feb 2016)

➢ In 2014, an unlicensed doctor in a remote area of Cambodia infected 300 people with HIV, due to contaminated needles. So far, 14 people have died, and others remain weakened from the infection. Cambodia has one of the world’s lowest doctor/patients ratios – 1 doctor per 5000 people – so unlicensed doctors are common. They are particularly common in remote areas with poor health infrastructure, and where it can be very expensive and difficult to travel to the nearest hospital. However, it can be argued that unlicensed doctors can help fill a yawning gap in provision, as they are on hand, can offer immediate help and are much more affordable – an important consideration when people may have to choose between food and medical care. Since the HIV outbreak, there has been a government clampdown on unlicensed doctors, many of whom do have medical training. Some NGOs are stepping in, by providing mobile clinics, training for unregistered midwives, and using local volunteers to share knowledge on health and nutrition. However, this does not resolve the fundamental problems of Cambodia’s health system being under–funded and under–resourced, which was highlighted by the HIV outbreak. (*Al Jazeera*, 11 Mar 2016)

➢ In March, Myanmar swore in its first civilian president for more than 50 years, and his party, Aung San Suu Kyi’s National League for Democracy won large majorities in both houses of parliament in November. Mr Thein Sein, the outgoing president, handed over power peacefully, and Mr Min Aung Hlaing, head of the army, openly supported Myanmar’s democratic transition. Whilst an apparent triumph for democracy, the situation is more complex, as Miss Suu Kyi (the people’s preferred choice for president) was barred from office by the constitution which disallows anyone with a foreign spouse or children from this role. Instead, she is in charge of the foreign ministry, and intends to run the country via the nominal president – installing a puppet president is an inauspicious start. However, the larger democratic threat is the power reserved for the army, which still controls defence, border affairs and home affairs – giving it control over the country’s entire administrative structure. It also dominates the National Defence and Security Council, which can impose martial law and run the country, and can potentially veto any constitutional changes. The country’s future success and prosperity depends on the civilian and army sides working together, despite controlling different parts of government. (*The Economist*, 3 Apr 2016)

➢ More than 57 000 Indonesian people with mental health conditions have been subjected to *pasung – *ie, being shackled or locked up in a confined space at least once, and currently 18 000 people are shackled. The practice was banned in 1977, but remains widespread due to a lack of awareness, superstition and inadequate mental health services (a law requiring mental health medication to be provided in primary health centres is not being implemented). In their report *Living in Hell*, the campaigning organisation Human Rights Watch calls on the government to enforce the ban on shackling and ensure access to mental health care. They recently met with Indonesia’s health minister, Nila Moeloek, and were encouraged by her commitment to providing mental health medication in 9 500 community health centres across Indonesia – which could help end shackling. They now call on her to demonstrate her willingness to deliver by giving a full plan for providing community health centres with the necessary medication – no longer should a lack of medication be used as an excuse to shackle people. (*Human Rights Watch*, 11 Apr 2016)

### AUSTRALIA AND WESTERN PACIFIC

➢ Tonga is the world’s most obese country, with up to 40% of the population estimated to have type 2 diabetes and other non–communicable diseases and life expectancies have fallen by 10 years (from the mid–70s to mid–60s). The traditional Tongan diet consisted of fish, root vegetables and coconuts, but gradually was replaced by cheap offcuts of meat from the USA and New Zealand and tinned produce. Tonga’s obesity problem may partly be genetic, as islanders had to survive long periods without food so their bodies are programmed to hold onto fat stores. However, Tongan society traditionally views overweight people as attractive, and there is a culture of over–eating at celebrations. There are government efforts to raise awareness of the risks of diabetes, but there is little evidence of changing lifestyles or diet. According to one doctor, this will take generations and things will worsen before they improve. (*BBC*, 18 Jan 2016)

➢ Australia’s annual *Closing the Gap* report shows that indigenous Australians continue to die younger, are more likely to be unemployed and with lower educational levels than other Australians. The government has seven targets across health, education and employment to close this gap, and there are five failings in these areas. First indigenous people die 10 years younger than non–indigenous people, although recent declines in mortality rates from chronic and circulatory diseases may improve this in the future. Second, the infant mortality rate for indigenous people is 6.4 per 1000, compared to 3.6 across all Australians. Third, 60% of indigenous students finish Year 12 of school, compared to 86.5% for other Australians. Fourthly, the employment rate amongst indigenous people is 47.5%, compared to 72.1% amongst non–indigenous Australians. Indigenous people are more vulnerable to economic shocks, and unlike non–indigenous Australians, their employment levels have fallen from 2008 levels. Finally, an indigenous man is 15 times more likely to be jailed than a non–indigenous man – indigenous people comprise 3% of the Australian population, but are 25% of the prison population. (*Sydney Morning Herald*, 10 Feb 2016)

➢ The Marshall Islands are the latest Pacific Island country to confirm the arrival of the zika virus. Although there is one sole case to date, the government has declared an “outbreak” and a state of health emergency due to the islands being extremely vulnerable to the disease. It is the islands’ dry season, and zika could spread further and affect more pregnant women when the rains start – the state of emergency aims to prevent this happening. Efforts are currently focused on pregnant women, with volunteers distributing leaflets house–to–house on cleaning, mosquito control, symptom recognition and zika kits (containing mosquito repellent, treated bed nets and condoms). The government is considering importing blood supplies to avoid transmission by contaminated blood. With scarce health facilities on the outlying islands, health officials are emphasising zika prevention in these areas. (*Radio New Zealand*, 8 Mar 2016)

➢ More than 200 000 women and girls in New Zealand have been vaccinated against the human papilloma virus (HPV), which is linked to several cancers, including cancer of the cervix and throat. However, in contrast to Australia where the vaccine is available to both males and females, New Zealand restricts state–funded HPV vaccinations to females, and males aged 9–26 who identify as gay. However, the prevalence of HPV–linked oropharyngeal cancers is higher amongst men – 4 per 100 000 compared to 1 per 100 000 for women – and is approaching the incidence of cervical cancer. This had led to campaigners to call for government funding for HPV vaccination to be extended to boys. “Men are more exposed to the virus because the route of exposure is understood to be oral sex and that the concentration of virus in the female genital tract is much higher than in the male tract,” says ENT surgeon Dr John Chaplin. (*New Zealand Herald*, 20 Mar 2016)

➢ A young woman – a refugee from Africa – was raped in Nauru’s refugee detention centre, and was sent to Papua New Guinea to terminate her pregnancy. Abortion is illegal in Papua New Guinea, and Australia’s Federal Court ruled that Australia owes her a duty of care to ensure she receives a safe and legal abortion – Australia’s government had previously argued that it owes no duty of care towards her. The woman – known only as S99 – suffers from violent epileptic seizures, severe mental health issues and specialist medical needs arising from a medical procedure she was subjected to as a young girl. Her barrister told the Court that sending S99 to Papua New Guinea for an abortion was tantamount to “procuring illegal conduct,” and the court ruled that it was unreasonable to consider an abortion procured in Papua New Guinea as a safe or legal procedure. However, the court did not require her to be brought to Australia for the procedure, and it is expected that a third country will be found for her termination. (*Sydney Morning Herald*, 6 May 2016)

## CHINA

➢ China’s government estimates that 13% of China’s mainland population has a “psychiatric handicap”, and the World Health Organization estimates it as almost 10%. Despite these large numbers, there are only 20 000 mental health specialists in the entire country, and 1.7 psychiatric beds per 10 000 people – the global average is 4.4 beds. This gap has led to Dr Guan Weili to set the Wenzhou Kangning Hospital, China’s largest private mental health facility. The hospital owns five centres, manages four others and plans to expand into Hong Kong. Although psychiatric hospitals have existed since 1949, the first mental health laws – including the right of patients not to be detained against their wishes – were only implemented in 2013. Mr Guan believes that the rise in psychiatric problems is linked to China’s rapid economic growth – indeed, some large corporations offer 24–hour helplines and suicide prevention counselling – comparing it to Japan at a similar stage of development. (*Forbes*, 26 Jan 2016)

➢ Shares in Alibaba Health Information Technology Ltd fell as China suspended a drug–coding system (PIATS) developed and owned by the company. PIATS identifies counterfeit medicines, and generated nearly 50% of the company’s revenue in the previous financial year. The company received no advance warning of the Chinese Food and Drug Administration’s decision, who have made draft amendments to existing rules to allow other methods of tracking drugs to their source. The company plans to continue operating PIATS, and will work closely with regulators to continue such operations under their direction. (*Bloomberg*, 22 Feb 2016)

➢ 27 people have been arrested for the illegal sale and distribution of incorrectly stored vaccines across China; and four hospital chiefs have been charged with buying vaccines from illegal sources. The vaccines are allegedly still safe, and people who have been vaccinated do not need to be re–vaccinated. However, neither the vaccines’ batch numbers, nor the clinics which have used them, have been disclosed, leading to parents and experts demanding more transparency. China’s State Council is reforming the legal framework for privately–produced vaccines by making provincial disease control centres responsible for purchasing vaccines. This would block the current loophole that allows vaccines to be sold privately. (*South China Morning Post*, 14 Apr 2016)

➢ 500 school students at the Changzhou Foreign Languages School in Jiangsu suffered serious health problems, including cancer, after their school was relocated to a site near former chemical plants. 493 students developed blood abnormalities, leukemia, lymphoma, dermatitis, eczema and bronchitis. A report on China’s state broadcaster China Central Television showed that soil and groundwater in the area contained toxic compounds and heavy metals, and one carcinogen was 100 000 times above the safety limit. It appears that the environmental assessment prior to the school’s construction did not look for pesticides, and builders used heavily–polluted groundwater during construction. China increasingly views environmental pollution as a destabilising social factor rather than the inevitable result of economic expansion, and has recently increased efforts to combat it. (*Irish Times*, 18 Apr 2016)

➢ Following the end of China’s one–child policy, the role of the country’s army of family planning officers is changing. Over the past 35 years, family planning officers covered cities, towns and villages across China, and uncovered families suspected of breaking China’s one–child legislation. They were widely distrusted – confiscating property if families couldn’t afford to pay fines levied for breaking the one–child rule, and pressurising women into abortions, even when women were six months’ pregnant. However, some are being retrained in child welfare, and are teaching parents and grandparents how to develop and stimulate toddlers’ minds by talking, singing and reading to them. Overall, China’s one–child policy has led to a shrinking labour–force that could undermine economic growth and innovation within the workplace, and investing in young children’s development could be essential for the country’s future. (*BBC*, 4 May 2016)

## EUROPE

➢ According to a report compiled by Health Consumer Powerhouse, waiting times for emergency treatment in Irish hospitals are the worst in Europe, and frequently exceed 3 hours. Overcrowding in emergency departments places the health system under strain, with a snapshot of 391 patients on trolleys showing that 210 had waited more than nine hours. In addition, waiting times for minor operations and CT scans also rank amongst Europe’s longest. Ireland also ranks poorly for direct access to a specialist and physical activity in schools, and worst for binge drinking. However, Ireland’s smoking rates are amongst Europe’s lowest, and is rated highly for access to essential drugs. Other problems include the high percentage of people purchasing duplicate health insurance, although the report praised Ireland’s “dedicated efforts” in halving the rate of MRSA infection between 2008 and 2015. (*Irish Times*, 26 Jan 2016)

➢ NGOs warn that HIV infections are rising in eastern Ukraine, where treatment and prevention programmes, condom distribution to high–risk groups, and needle exchange programmes have been affected by two years of conflict between pro–Russian separatists and government forces. Even before the conflict, Ukraine had one of the highest rates of HIV infection in Europe – 1.2% of Ukrainians aged between 15–49 years were HIV–positive, and this may have doubled. 30% of new infections are in the partially rebel–controlled areas of Donetsk and Luhansk, and HIV–related tuberculosis infections are also common. There are shortages of antiretroviral treatment – although this is slowly improving – and diagnostic and treatment equipment. Mikhail, an HIV–positive man, says “it’s hard to live in such conditions. Half of the pharmacies are closed [and those that are open] don’t even have the most basic medication.” (*IRIN*, 23 Feb 2016)

➢ Despite high overall spending on welfare, Finland’s health system is under–resourced, with long waiting times – a 2012 report found that 80% of people had to wait 2 weeks to see a family doctor – and severe cost pressures. Finland spends 7% of its GDP on health, compared to 8% in the UK, and has a high proportion of private primary care doctors. Some Finnish people use private doctors in nearby Estonia to save time and money. The health service is locally funded, so that poorer areas have correspondingly lower–quality health care. Users of the public health system face high charges for drugs and consultations, and many people are forced to use private health care. However, reforms are planned, with 301 municipalities being merged into 19 larger, more efficient, organisations. (*The Guardian*, 23 Feb 2016)

➢ In the country’s 2016 Budget, the UK’s Chancellor, Mr George Osbourne, announced a sugar tax on soft drinks. The tax, aimed at reducing the rise in childhood obesity, will begin in 2018 and money raised will fund sports activities in primary schools. It will be levied on drinks companies, and assessed on the volume of sugar–sweetened drinks they produce or export. The tax will be graduated, with one level for drinks with sugar above 5 g per 100 ml, and a second level for drinks with more than 8 g per 100ml, leading to price increases of GBP 0.08 (US$ 0.11) per drink. Mr Osbourne noted that a can of cola typically contains 9 teaspoons of sugar, and others have up to 13 teaspoons – more than double a child’s recommended added sugar intake. A 2015 report by Public Health England recommended a 10–20% tax on high–sugar products. The move was welcomed by the British Medical Association, the Labour Party and Jamie Oliver, the celebrity chef and healthy eating campaigner, although the political party UKIP opposed it. (*Huffington Post*, 16 Mar 2016)

➢ Romania’s health minister Patriciu Achimas–Cardariu has resigned following public protests over the use of sub–standard disinfectant in dozens of Romanian hospitals. Authorities conducted searches at hospitals and at the drug company, Hexi, which reportedly supplied the disinfectant for use on surfaces and hands. Police also took away documents and samples from 25 hospitals. This is the latest problem to hit Romania’s underfunded health system, which faces an outflow of staff and systemic bribery and informal payments. (*Washington Post*, 9 May 2016)

## INDIA

➢ A 2010 World Bank estimate showed that premature mortality, lost productivity, health care provision and other losses due to inadequate sanitation costs India US$ 53.8 billion each year. However, Shamika Ravi and Rahul Ahluwalia argue that large gains in India’s public health, especially the health of its poorest people, can be made if the government prioritises the expansion and effective delivery of “public goods”, such as vaccination, health education, sanitation, public health, primary care and screening, and reproductive and child health. These gains could be made economically, as neighbouring Sri Lanka and Bangladesh spend less on health as a percentage of GDP, yet have better outcomes. India should therefore focus on setting appropriate goals, and reforming the public health sector’s governance and management systems so it can deliver these goals. There are also severe shortages of qualified health professionals, especially in rural areas, and the government must be creative in addressing this. Health financing requires reform, and Shamika Ravi and Rahul Ahluwalia also urge the adoption of Medical Savings Accounts with tax deductions for medical expenses, and direct payments for those who cannot pay themselves. (*Brookings*, 26 Jan 2016)

➢ New Delhi’s pollution levels exceeded Beijing and Shanghai on 24 Dec 2015, when they reached levels of 295mg/m^3^ for PM 2.5 and 470 mg/m^3^ for PM 10 (against recommended upper limit of 60 and 100 respectively). The city’s pollution crisis has led to a sharp rise in respiratory illnesses, skin and eye allergies, cardiac arrest, memory loss, depression and lung damage, and 4–in–10 children suffer from severe lung problems. Pollution is responsible for 10 000–30 000 deaths in New Delhi each year, and is the 5^th^ largest cause of death in India. New Delhi’s massive population growth is fuelled by polluting industries, sharp rises in the number of vehicles, and spiralling energy consumption supplied by polluting power stations. Thanks to public pressure, the Delhi government has introduced a number of measures, including road rationing to reduce vehicle pollution. Environmentalists state that this ignores the role of industry, and calls on Delhi to copy China’s lead in issuing pollution–related alerts. (*The Diplomat*, 8 Jan 2016)

➢ India is conducting its first survey of the prevalence of drug–resistant tuberculosis (TB), and plans to release the results by December 2016. With an estimated 2.1 million cases, India has the world’s highest number of TB patients, and is believed to have the largest number of drug–resistant TB cases after China. The World Health Organization describes drug–resistant TB as a global threat to TB treatment. The survey would improve the detection of drug–resistant TB by highlighting high–prevalence areas, and inform India’s future TB–control strategy – and improved detection is vital to prevent transmission of the disease. (*Reuters*, 7 Mar 2016)

➢ Diabetes is rapidly increasing in India, with 70 million cases amongst the adult population in 2015, and prevalence has risen by 80% amongst women between 1980 and 2014. According to research published in the *Lancet,* India has the second–highest prevalence of diabetes worldwide, and until it was recently overtaken by China its prevalence rate was the highest. To help alleviate the problem, India’s National Programme for Prevention and Control of Cancer, Diabetes, Cardiovascular Diseases and Stroke is focusing on awareness raising, behaviour and lifestyle changes, screening, and early diagnosis and referral for at–risk people. (*Times of India*, 27 Apr 2016)

➢ In 2015, India inaugurated its Mission Indrahanush, which aims to immunise every child against 7 vaccine–preventable diseases by 2020, and achieve above 90% coverage – compared to the current 65%. This target is additionally ambitious, in light of India’s Universal Immunisation Programme (UIP) introduction of four new vaccines (polio, rotavirus, rubella and Japanese encephalitis). However, there are 25 vaccine–preventable diseases, which will require the expansion of the UIP over time, raising the issue of funding. This is particularly complex given India’s large population, and it will be ineligible for GAVI support by 2021. A recent report published by the IKP Trust and Global Health Strategies outlined the possible costs and finance options for expanded immunisation coverage. Amongst other options, it considered a “National Trust Fund for Health and Immunisation.” Self–financing trust funds can potentially protect against volatility in drug prices and donor support. The report authors also call for all expenditure on maternal and child health and preventative health care to be re–classified as capital expenditure rather than revenue expenditure; and for the US$ 471 million needed for India to meet its immunisation targets to be allocated additionally and separately from the health ministry budget and ring–fenced as an annual recurring outlay, indexed for inflation. (*Asian Age*, 6 May 2016)

## THE AMERICAS

➢ Barbados was one of seven countries to take part in the first WHO/UN Development Programme global project on adapting public health systems to climate change. In Barbados, one of the key aims was to improve water storage facilities to eliminate mosquitoes, give technical advice on building and maintaining water tanks, and raise awareness about safe ways to harvest rainwater. This is especially crucial for Barbados, which has a high rate of dengue fever and some recently–detected cases of zika. As zika spreads, there is more pressure to analyse the health impacts of climate change and extreme weather, and to understand how climate stresses can shape health risks. Although any link between climate change, powerful El Niño weather phenomenon and the rise of zika is unproven, it is certainly plausible that unusual weather conditions make it easier to transmit the virus. Understanding these linkable could lead to targeted preventative public health measures in areas at high risk of an outbreak. (*Thomson Reuters Foundation*, 29 Feb 2016)

➢ According to a report from Amnesty International, access to contraception and abortion can be a lottery in Latin America, and is often dependent on the woman’s ability to pay or the personal and religious views of health workers. In most countries in the region, abortion is only allowed in cases of rape, incest or if the mother’s life is in danger, giving the region some of the world’s strictest abortion laws. When combined with the low availability of contraception – according to the UN Population Fund, 1–in–3 women of child–bearing age who would like to use birth control has no access – women can be forced to undergo dangerous backstreet abortions, which causes at least 10% of maternal deaths in Latin America. Each year, around 760 000 women in Latin America receive hospital treatment for complications related to unsafe abortions. This spread of zika has led to some countries recommending that women delay pregnancy. However, Amnesty states “this recommendation is not just absurd, it is insulting in a region where more than half of pregnancies are unwanted or unplanned, where there are extremely high rates of sexual violence, where the demand for contraception far outstrips availability.” (*Thomson Reuters Foundation*, 7 Mar 2016)

➢ Almost 10% – US$ 619 billion – of the US government’s health spending is on Medicare, the country’s health insurance scheme for elderly people. Medicare covers the average market price for a drug, plus a 6% premium and separate compensation for administering the drug. This incentivises doctors to prescribe expensive drugs over cheaper, similar, drugs. To reduce costs, the federal government has proposed reducing the 6% premium to 2.5%, plus a flat fee for treatment. There are concerns that this proposal will stifle innovation from smaller providers, as lower margins could mean that treatment could only be provided at scale, eg, within hospitals. Doctors and drugs companies, the main beneficiaries of the current arrangements, are also strongly opposed to the proposal, but it is difficult to disentangle justifiable concerns from scaremongering. (*The Economist*, 16 Apr 2016)

➢ Following the 7.8 magnitude earthquake which struck Ecuador in May, killing more than 600 people, causing billions of dollars’ worth of damage and leaving 720 000 people in need of humanitarian assistance, the government and the UN have launched an urgent appeal for US$ 72.7 million from the international community. However, response has been slow with donors only raising US$8.6 million, with an additional US$ 6.5 million pledged outwith this appeal. This brings the total to US$ 15.1 million – far short of what is required. Homes, roads and public infrastructure have been razed by the earthquake, and Ecuador’s already–struggling economy will slip back into recession as GDP will fall by an estimated 2–3%. Lenders such as the World Bank have opened US$ 600 million of credit to help the Ecuador response, and the government announced emergency finance measures (one–off taxes, asset sales etc.) to fund reconstruction. “We thank those countries who have responded to our appeal and call on others to do the same,” said Mr Jens Laerke, a UN spokesperson. (*Public Finance International*, 9 May 2016)

➢ Undocumented immigrants in Canada often avoid mainstream health services because they fear being reported to the country’s border services – some patients have said they could rather die than be deported – and the US$ 695 hospital consultation fee acts as a further deterrent. Bryon Cruz, an outreach worker with the migrants’ rights group Sanctuary City, works to connect undocumented immigrants with the health care they need, and notes that people have been deported for accessing medical services. He receives 25 calls a week from undocumented immigrants too afraid to access mainstream services, and he has a network of social and health care workers who will treat these people without reporting them. Examples of this work include an off–duty doctor replacing a patient’s dislocated shoulder behind a café, and a veterinarian was on the brink of stitching another patient’s wound until a nurse came forward. Mr Cruz has seen progress from Vancouver Coastal Health and Fraser Health on not reporting immigrants, and calls for the children of undocumented immigrants to get free health care. (*CBC news*, 5 May 2016)

